# CKD-497 inhibits NF-kB signaling and ameliorates inflammation and pulmonary fibrosis in ovalbumin-induced asthma and particulate matter-induced airway inflammatory diseases

**DOI:** 10.3389/fphar.2024.1428567

**Published:** 2024-08-07

**Authors:** Hyejeong Kim, Jihye Choi, Jaeok Seo, Hyungjoon Lim, Sung Kwon Kang

**Affiliations:** Department of Synthetic Chemistry, Chong Kun Dang Research Institute, Yongin-si, Gyeonggi-do, Republic of Korea

**Keywords:** ovalbumin, asthma, fine dust, airway inflammation, functional food, respiratory disease

## Abstract

**Introduction:** Air pollution, allergens, and bacterial infections are major contributors to pathological respiratory disorders worldwide. CKD-497, derived from the rhizome of *Atractylodes japonica* and the fruits of *Schisandra chinensis*, is known for its ability to relieve cough and facilitate phlegm expectoration. However, its protective action against allergic asthma and fine dust-induced lung inflammation, along with its underlying mechanisms, have not been thoroughly investigated.

**Methods:** In this study, we established mouse models of ovalbumin (OVA)-induced asthma and particulate matter (PM)-induced pulmonary inflammation to evaluate the effects of CKD-497. Mice were administered CKD-497 orally, and various parameters such as airway inflammation, mucus production, and proinflammatory cytokine levels (IL-1β, IL-6, TNF-α) were measured. Additionally, the macrophage cell line RAW264.7 was pretreated with CKD-497 and stimulated with lipopolysaccharide (LPS) to assess inflammation via the NF-kB signaling pathway.

**Results:** Oral administration of CKD-497 effectively attenuated airway inflammation and mucus production in both OVA-induced asthma and PM-induced lung inflammation models. It also significantly decreased the production of proinflammatory cytokines IL-1β, IL-6, and TNF-α. CKD-497 alleviated leukocyte infiltration, including neutrophils, and reduced fibrillary collagen deposition in PM_10_-treated mice. *In vitro*, CKD-497 pretreatment inhibited LPS-induced inflammation in RAW264.7 cells through the suppression of the NF-kB signaling pathway.

**Discussion:** CKD-497 shows potent anti-inflammatory effects in mouse models of asthma and PM-induced lung inflammation, potentially mediated by the inhibition of the NF-kB pathway. These findings suggest that CKD-497 could serve as a functional supplement to protect against respiratory diseases by mitigating pulmonary and airway inflammation induced by allergens and air pollution.

## 1 Introduction

Asthma is a chronic respiratory disease affecting one-third of the world’s population and causing 2.5 million deaths annually (Rehman et al., 2018; Stern et al., 2020). It is characterized by airway inflammation triggering smooth muscle contraction, microvascular leakage, and airway mucus secretion, resulting in narrowing of the airway and obstruction (Barnes, 2008). Repetitive exposure to various stimuli, such as the inhalation of allergens (dust particles, pollen, and air pollutants), has been reported to exacerbate asthma and chronic obstructive pulmonary disorders (Sim et al., 2014; Jiang et al., 2016). Notably, long-term exposure to fine dust increases the risk of developing asthma and promotes the development of respiratory diseases by inducing persistent lung inflammation (Tiotiu et al., 2020). Particulate matter (PM) inhalation causes immune cell infiltration into the alveoli, which then release inflammatory cytokines (Boorsma et al., 2013; Wang et al., 2017). The released inflammatory cytokines disrupt pulmonary epithelial integrity, resulting in bronchial wall thickening and impaired gas exchange (Butt et al., 2016; Thangavel et al., 2022; Kim et al., 2023). Recent studies have reported that dietary components hold the potential as promising therapeutics for treating asthma and respiratory diseases. For example, Gingerols, shogaols, and their metabolites derived from ginger root relax precontracted human airway smooth muscle (Zhang et al., 2022). A fatty acid-rich fraction of sea cucumber extract ameliorated allergic airway inflammation (Lee et al., 2018). In addition, *Artemisia argyi* attenuates the elevation of allergic responses and mucus overproduction in ovalbumin (OVA)-challenged asthmatic animals (Shin et al., 2017).

CKD-497, an herbal formula extract obtained from the rhizome of *Atractylodes japonica* and the fruits of *Schisandra chinensis*, relieves coughing and facilitates the expectoration of phlegm by enhancing mucociliary clearance and reducing mucin viscosity (Chae et al., 2020). However, the protective action of CKD-497 against allergic asthma and fine dust-induced lung inflammation, as well as its underlying mechanism, remain unclear. In the present study, we aimed to investigate the therapeutic potential and anti-inflammatory molecular mechanisms of action of CKD-497 in an *in vitro* cell model of lipopolysaccharide (LPS)-induced inflammation and in *in vivo* mouse models of ovalbumin-induced asthma and fine dust-induced lung inflammation. Our results suggest CKD-497 as a potential functional supplement that can protect against respiratory diseases by inhibiting pulmonary and airway inflammation induced by allergens and air pollution.

## 2 Materials and methods

### 2.1 Materials

CKD-497 is a new plant-based functional food obtained from the CKD Pharmaceutical Company (Yongin-si, Gyeonggi-do, Republic of Korea). A mixture of the dried rhizome of *A. japonica* and the fruit of *S. chinensis* (5:1 weight ratio) was extracted at approximately 1°C–30°C via immersion with an 8-fold volume of 50% ethanol for 24 h. This extraction process was repeated using a 6-fold volume of 50% ethanol, and the solution was filtered using a 4 μm thick paper. The extracted solution was then concentrated under reduced pressure below 60°C to obtain the soft extract of CKD-497. Gas chromatography analysis revealed that CKD-497 contained 72 ppm (mg/kg) of residual ethanol solvent, which was a negligible amount.

### 2.2 Cell culture

RAW 264.7, a mouse macrophage cell line, was purchased from American Type Culture Collection (TIB-71™, Manassas, VA, United States) and cultured in high glucose Dulbecco’s modified Eagle’s medium (DMEM) (#11965092; Gibco, Thermo Fisher Scientific, Waltham, MA, United States) supplemented with 10% heat-inactivated fetal bovine serum (FBS, #A3160501; Gibco), 300 mg/L L-glutamine, 25 mM HEPES, 25 mM NaHCO_3_, and 1% penicillin/streptomycin (#0503; Gibco). The cells were routinely passaged at 60%–70% confluence and maintained at 37 °C in a humidified atmosphere containing 5% CO_2_.

### 2.3 Western blot analysis

RAW 264.7 cells were seeded in 6-well plates at the density of 1 × 10^6^ cells/well and incubated overnight at 37°C in 10% DMEM. The following day, the cells were starved of media for 2 h and then pretreated with CKD-497 (100 and 200 μg/mL) for 2 h, followed by a 2 h incubation with LPS (#L2880; Sigma-Aldrich, St. Louis, MO, United States). Cells were subsequently harvested, and proteins from the cell lysates were extracted in radioimmunoprecipitation assay buffer (100 mM Tris-Cl, 5 mM EDTA, 50 mM NaCl, 50 mM β-glycerophosphate, 50 mM NaF, 0.1 mM Na_3_VO_4_, 0.5% NP-40, 1% Triton X-100, and 0.5% sodium deoxycholate). Sample protein concentrations were quantified using a Quick Start™ Bradford protein assay kit 2 (#5000202; Bio-Rad Laboratories, Hercules, CA, United States). Subsequently, the cell lysates were separated using sodium dodecyl sulfate-polyacrylamide gel electrophoresis and transferred onto nitrocellulose membranes. The membranes were blocked using 3% bovine serum albumin in 0.1% Tris-buffered saline containing Tween 20 and probed using primary antibodies. The following primary antibodies were obtained from Cell Signaling Technology (CST, Danvers, MA, United States) and were used at 1:1,000 dilution: NF-κB p65 (#8242), phospho-NF-κB p65 (#3031), phosphor-IκBα (#2859), IκBα (#4814), and β-actin (1:2,000, #4967). The membranes were then incubated with horseradish peroxidase-conjugated goat anti-rabbit IgG or goat anti-mouse IgG (#31,460 and #31,430, respectively; Thermo Fisher Scientific). β-actin was used as the loading control.

### 2.4 Animals and experimental design

#### 2.4.1 OVA-induced mouse model of asthma

**FIGURE 1:**
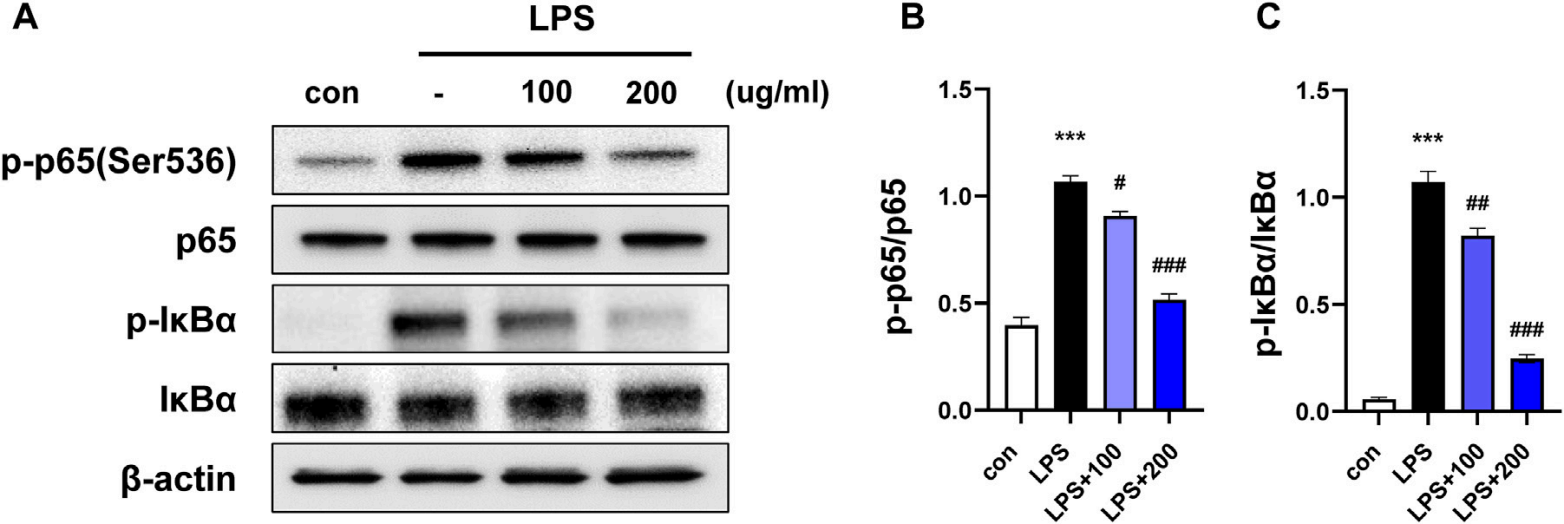
CKD-497 inhibits NF-κB signaling in LPS-induced RAW 264.7 cells. **(A)** RAW 264.7 cells were pretreated with CKD-497 for 2 h and treated with LPS for 2 h. Expression levels of phospho-p65 NF-κB protein (p-p65), p65, phospho-IkBα and IkBα was analyzed using Western blotting; β-actin used as the loading control. **(B–C)** Quantitative graph of Western blot analysis using ImageJ software as p-p65 per p65 and p-IkBα per IkBα. All data were analyzed using a one-way analysis of variance followed by Dunnett’s multiple comparison test. ^***^ *p* < 0.001 vs. control. ^###^ *p* < 0.001, ^##^ *p* < 0.01, ^#^ *p* < 0.05 vs. LPS. Results are presented as mean ± SEM values.

Five-week-old male C57BL/6NHsd mice were purchased from Koatech (Gyeonggi-do, South Korea). All mice were housed under controlled conditions (23°C ± 3°C, 12-h light/dark cycles, 55% ± 15% humidity, and specific pathogen-free conditions) and provided free access to food and water. All animal experiments were approved by the Institutional Animal Care and Use Committee of KNOTUS (approval number: KNOTUS IACUC 20-KE-713), Korea. All animal experiments were performed in accordance with the animal research: reporting of *in vivo* experiments (ARRIVE) guidelines. After a 7 day acclimation period, mice were sensitized and challenged with OVA as described previously. The mice were randomly divided into four groups (n = 10/group): control, OVA, and OVA + CKD-497 (two groups, one receiving a dose of 100 mg/kg body weight and the other 200 mg/kg body weight) and sensitized on day 0 and 14 via intraperitoneal injection of 75 μg OVA/100 μL Dulbecco’s phosphate-buffered saline (DPBS) (#D8537; Sigma-Aldrich). The residual ethanol content of CKD-497 was determined to be negligible (<0.014%); therefore, a separate vehicle group containing ethanol was not established. Mice were then challenged via intranasal instillation of 50 μg OVA/30 μL DPBS three times per week for 3 weeks (days 21–42). CKD-497-treated mice were orally administered 100 and 200 mg/kg body weight of the extract three times per week for 3 weeks (day 21˗42) (Figure 1A) and euthanized on day 42.

#### 2.4.2 PM_10_-induced mouse model of lung inflammation

Six-week-old female BALB/c mice were purchased from Koatech and housed under controlled conditions (23°C ± 3°C, 12 h light/dark cycles, 55% ± 15% humidity, and specific pathogen-free conditions); they were provided free access to food and water. All animal experiments were approved by the Institutional Animal Care and Use Committee of KNOTUS (approval number: KNOTUS IACUC 20-KE-711), Korea, and were performed in accordance with the guidelines and regulations for the animal care committee. Standardized and characterized PM_10_ (urban dust, 10 μm pore size) was purchased from the National Institute for Standards and Technology (NIST; #SRM 1648a; Gaithersburg, MD, United States). PM_10_ is defined as inhalable particles with a diameter <10 μm according to the United States Environmental Protection Agency. After the 7 day acclimation period, mice were randomly divided into four groups: control (treated only with PBS), PM_10_, and PM_10_ + CKD-497 (at doses of 100 and 200 mg/kg body weight). To establish the murine model of PM_10_-induced lung inflammation (PM_10_ model), anesthesia using a mixture of 2.5% isoflurane (Baxtor, Deerfield, IL, United States) in 33% oxygen and 67% nitrous oxide was administrated to each mouse via facemask. After anesthesia, the front teeth of the mice were fixed, and 20 μL of a PM_10_ solution was injected into the airways using a 24G catheter in a 1 mL syringe. PM_10_-treated mice were intratracheally administered 200 μg PM_10_ dissolved in 20 μL PBS (days 0, 1, 7, 8, 14, and 15), and the control mice were intranasally treated with 20 μL PBS (days 0, 1, 7, 8, 14, and 15). PM_10_ + CKD-497 100 mg/kg and PM_10_ + CKD-497 200 mg/kg were administered orally once a day for 9 days (day 1–9) using a Zonde needle (0.3 mL/head).

#### 2.4.3 Collection of serum and bronchoalveolar lavage fluid (BALF)

For serum collection, each mouse was anesthetized using avertin (240 mg/kg), and whole blood was collected from the abdominal vena cava using a 1 mL syringe. The collected blood was allowed to clot at room temperature for 20 min. The samples were then centrifuged at 201 × *g* for 10 min at 4°C, and the supernatant was collected. BALF samples were collected according to a previously reported method (Jin et al., 2017). Briefly, after an incision was made to expose the tracheal bronchus, the right bronchus was ligated using 3-0 silk. A catheter was then inserted into the left trachea, and the lungs were washed three times using 1 mL PBS.

#### 2.4.4 Measurement of cytokine levels in serum

The levels of inflammatory cytokines were measured in serum samples. The levels of interleukin (IL)-6, IL-1β, IL-17A, and tumor necrosis factor (TNF)-α were measured using Quantikine enzyme-linked immunosorbent assay (ELISA) kits (#MLB00C, #MTA00B, #M6000B, and #M1700, respectively R&D systems, Minneapolis, MN, United States), following the manufacturer’s instructions.

### 2.5 Differential cell counting using fluorescence-activated cell sorting (FACS)

To determine the number of each cell type in the BALF sample, BALF cells were resuspended in FACS buffer (PBS containing 0.5% BSA, pH 7.2) and stained with different antibody cocktails to identify macrophages, lymphocytes, and neutrophils, followed by FACS analysis. The cells were incubated with monoclonal antibodies against CD45, Ly6G, CD11b, CD11c, SiglecF, and F4/80 (#63-0451-82, #11-9668-82, #47-0112-82, #45-0114-82, #50-1702-82, and #25-4801-82, respectively; Invitrogen, Carlsbad, CA, United States). Thereafter, they were washed with PBS, fixed with 0.5% paraformaldehyde for 20 min, washed again with PBS, and analyzed using two-color flow cytometry on FACSCalibur. The CellQuest™ Pro software was used for data analysis (BD Biosciences, Franklin Lakes, NJ, United States).

### 2.6 Histological and immunohistochemical analysis in OVA-induced mouse model of asthma

Lung tissue sections were stained using hematoxylin-eosin and Masson’s trichrome stains. To determine the severity of inflammatory cell infiltration, the peribronchial cell count, mucus production, and goblet cell hyperplasia were quantified in the airway epithelium in a blinded manner using a 4-point (0–3) grading system.

### 2.7 Quantification of lung fibrosis in PM_10_-induced mouse model of inflammation

The lungs of mice were fixed using 4% paraformaldehyde in PBS at 4°C for 48 h. After embedding in paraffin, 5 μm sections were prepared and stained with hematoxylin and eosin (HE). To quantify the collagen content in the lungs, the sections were stained using a Picro-Sirius Red kit (#ab150681; Abcam, Cambridge, United Kingdom). Images were obtained using an optical microscope (BX53; Olympus, Tokyo, Japan), and the Sirius Red-positive areas were quantified using the ImageJ software 1.40 (National Institutes of Health, Bethesda, MD, United States), and the area of the collagen deposition was expressed as a percentage of the whole lung area.

### 2.8 Statistical analysis

GraphPad Prism 10.0 (GraphPad Software, Inc., La Jolla, CA, United States) was used for data analysis. The results are presented as mean ± standard deviation (SD). The data were analyzed using one-way analysis of variance, followed by Dunnett’s multiple comparison test. *p* < 0.05 was considered statistically significant.

## 3 Results

### 3.1 CKD-497 inhibits LPS-induced NF- κB signaling in RAW 264.7 cells

LPS induces an inflammatory response, and the expression of inflammatory enzymes and cytokines is regulated via the nuclear factor-kappa B (NF-κB) signaling pathway (Yuan et al., 2013). To confirm whether the anti-inflammatory properties of CKD-497 were mediated by the NF-κB signaling pathway, RAW 264.7 cells were preincubated with CKD-497 (100 and 200 μg/mL) for 2 h before LPS treatment (100 nM). The expression of p-p65 and p-IκBa was significantly enhanced in the LPS-treated compared to that in the control group. Notably, this LPS-induced upregulated expression of p-p65 and p-IκBa was significantly inhibited in the CKD-497 200 μg/mL group compared to that in the LPS-treated group (Figures 1A–C). These results demonstrate that CKD-497 prevented inflammatory responses in LPS-induced RAW 264.7 macrophages by inhibiting NF-κB activation.

### 3.2 CKD-497 attenuates OVA-induced pulmonary damage and airway inflammation

**FIGURE 2:**
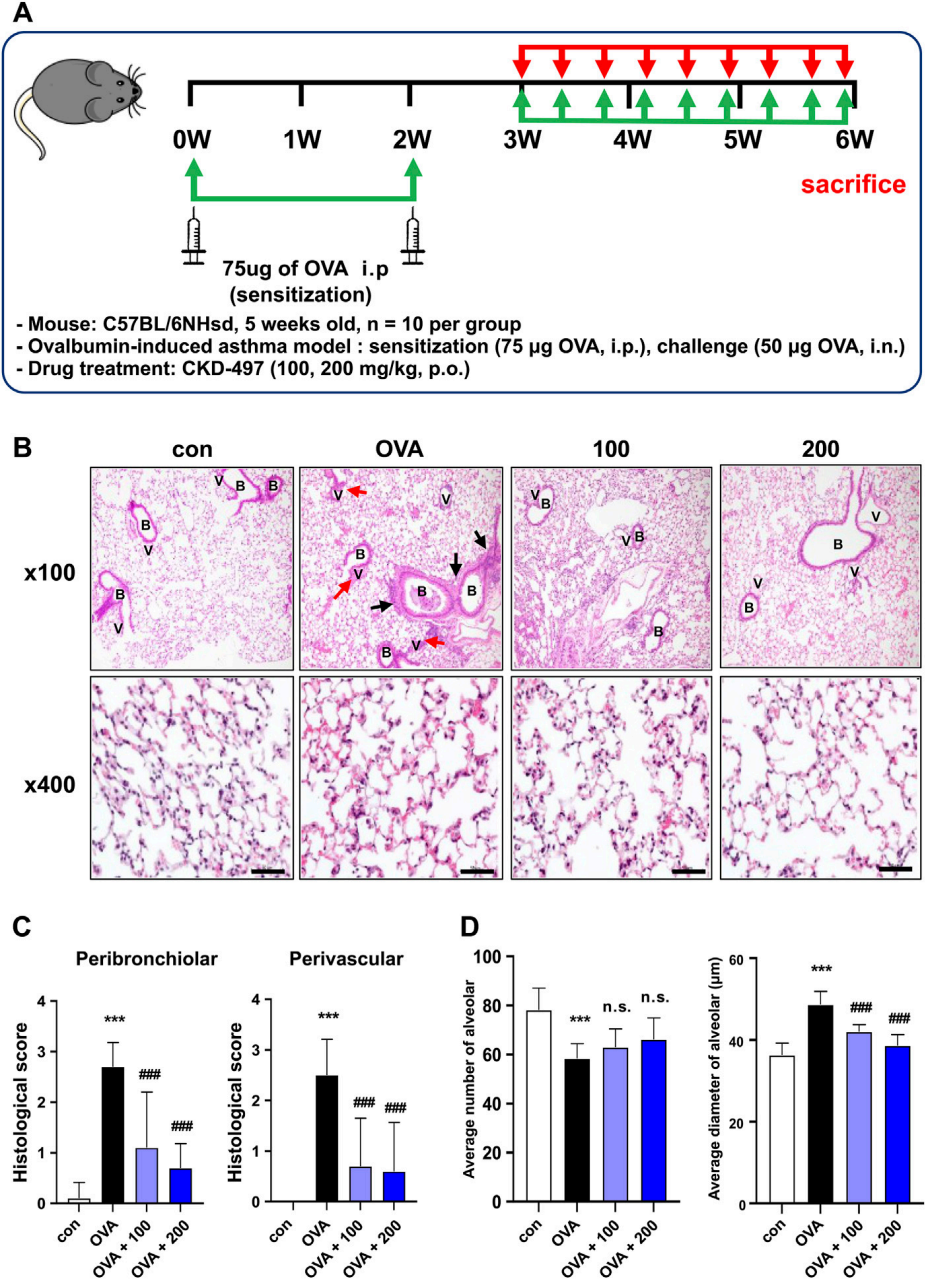
CKD-497 attenuates airway inflammation and remodeling in an OVA-induced mouse model of asthma. **(A)** Experimental scheme of the allergic asthma model. Mice were sensitized by OVA on weeks 0 and 2. Mice were treated with 50 μg of OVA for a period of 3 weeks (3 times per week, approximately 1 h before administration of the test substance) starting from week 3 (day 21). **(B)** Representative hematoxylin-eosin-stained images of the sections from mouse lungs. Lung injury was indicated by infiltration of immune cells, interstitial hemorrhage (black arrows), and perivascular hemorrhage (red arrows). Magnification, ×100 (upper panel) and ×400 (lower panel). Scale bar: 50 μm. **(C)** Histological scores were evaluated according to the grades as described in the Materials and methods. All data were analyzed using a one-way analysis of variance followed by Dunnett’s multiple comparison test. ^***^ *p* < 0.001 vs. control. ^###^ *p* < 0.001 vs. PM_10_. Results are presented as mean ± SD values. **(D)** The average alveolar size and diameter were quantified. n = 10 per group. OVA, ovalbumin.

OVA is widely used as an antigen to induce IgE-mediated allergic asthma in mice (Kim et al., 2019). To investigate the effect of CKD-497 *in vivo* in an OVA-induced allergic model of asthma, mice were intraperitoneally injected with OVA (Figure 2A). OVA-challenged mice exhibited typical features of asthma, including airway inflammation and airway hyperresponsiveness (Figure 2B). H&E staining revealed that the OVA challenge induced a marked infiltration of inflammatory cells into the peribronchiolar and perivascular regions of the lung tissue, which was strongly attenuated by treatment with CKD-497 (Figures 2B,C). In addition, OVA-induced allergic reactions caused lung inflammation, resulting in a decreased average number of alveoli and an increased diameter of the alveoli. Moreover, significant amelioration of alveolar wall thickening was observed in the OVA + CKD-497 (100 and 200 mg/kg) groups. However, CKD-497 administration, at both 100 and 200 mg/kg, exhibited no effect on OVA-induced decrease in the average number of alveoli (Figure 2D). These results demonstrate that CKD-497 exerts a protective effect against OVA-induced lung damage by inhibiting airway inflammation.

### 3.3 CKD-497 inhibits goblet cell hyperplasia in lung tissue

**FIGURE 3:**
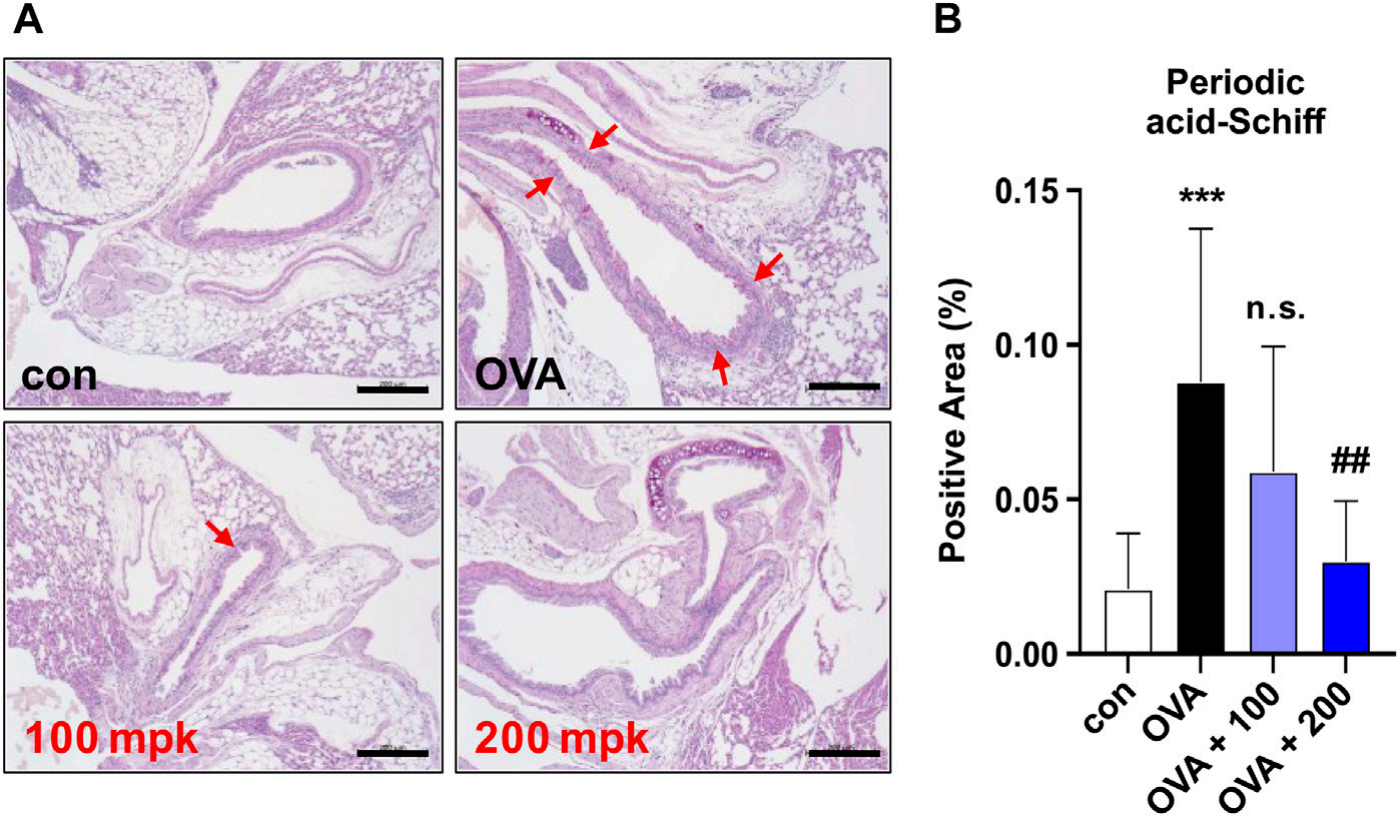
Effect of CKD-497 on goblet cell hyperplasia in allergic airway epithelia. **(A)** Periodic acid-Schiff (PAS) staining. PAS staining showed that the number of mucin-containing epithelial cells was significantly higher in the OVA-treated groups than in the control. Red arrows indicate PAS-positive cells. **(B)** The percentage of positive areas was calculated by dividing the PAS-positive area by the total area. Scale bar: 200 μm. All data were analyzed using a one-way analysis of variance followed by Dunnett’s multiple comparison test. ^***^ *p* < 0.001 vs. control. ^##^ *p* < 0.01 vs. PM_10_.

Airway goblet cell hyperplasia is one of the earliest hallmarks of airway remodeling in the OVA-treated mouse model (Zeki et al., 2010). To evaluate airway hypersecretion of mucus and goblet cell hyperplasia, mice lung sections were stained with periodic acid-Schiff base (PAS). The percentage of the PAS-positive areas (%) was significantly higher in the OVA-treated group than in the control group. Goblet cell hyperplasia in the airway was clearly observed in OVA-challenged mice compared to control mice. PAS staining revealed that treatment with CKD-497 at 200 mg/kg markedly inhibited goblet cell hyperplasia and mucus overproduction in the trachea and bronchiolar airways of OVA-challenged mice (Figures 3A,B). These results indicate that CKD-497 attenuates mucus production by inhibiting hyperplasia of goblet cells in the airway epithelium.

### 3.4 Effect of CKD-497 on total inflammatory, differential cell count, and inflammatory cytokine profiles

**FIGURE 4:**
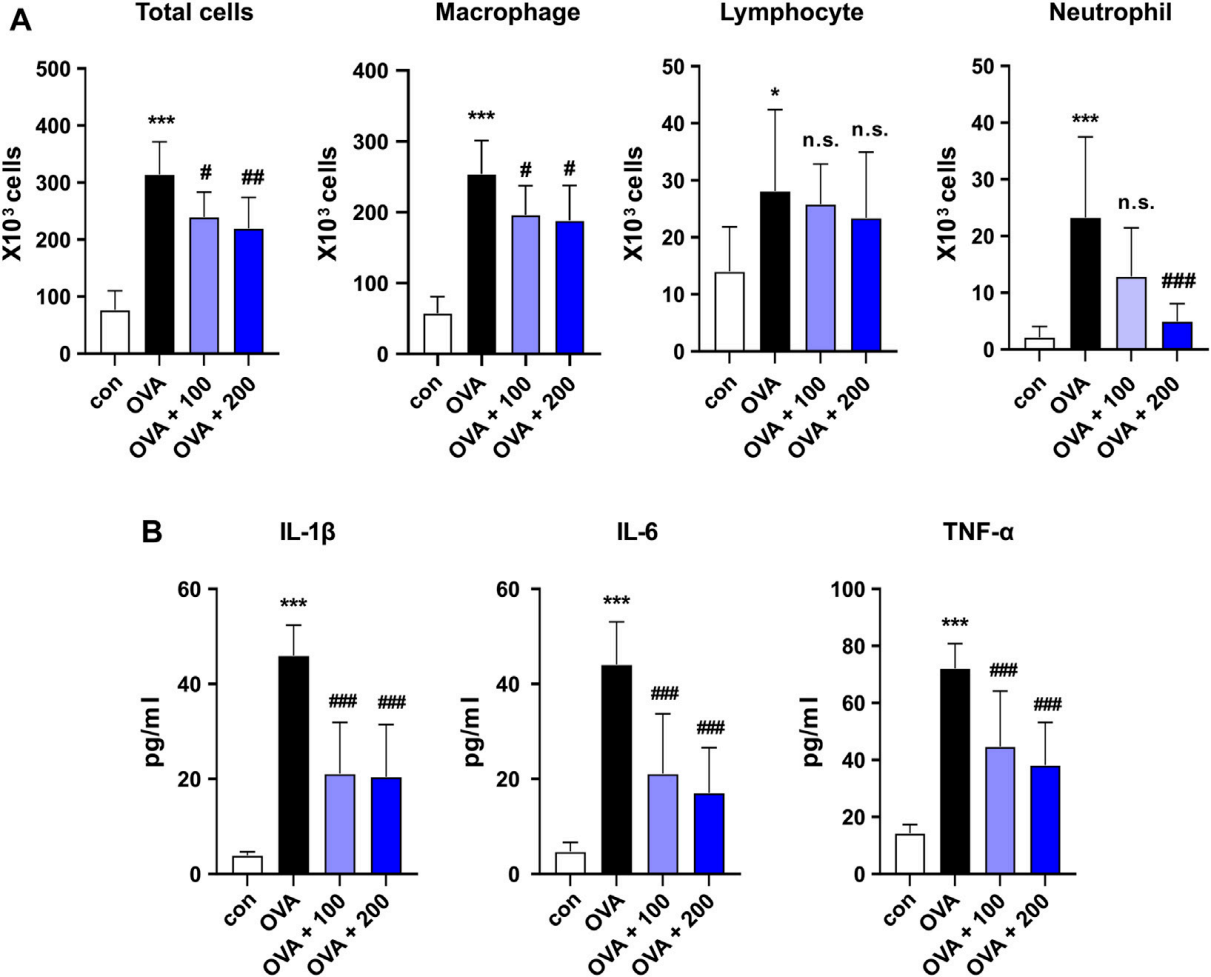
Effect of CKD-497 on the levels of immune cells and cytokine production in OVA-stimulated mouse model of asthma. **(A)** Total cells in BALF were counted in the images using a hemocytometer. Macrophages, lymphocytes, and neutrophils in the BALF were analyzed by flow cytometry. **(B)** IL-1β, IL-6, and TNF-α levels in serum were measured using ELISA. All data were analyzed using a one-way analysis of variance, followed by Dunnett’s multiple comparison test. ^***^ *p* < 0.001 vs. control. ^###^ *p* < 0.001, ^##^ *p* < 0.01, ^#^ *p* < 0.05 vs. OVA. Results are presented as mean ± SD values. OVA, ovalbumin; BALF, bronchoalveolar lavage fluid; IL-1β, interleukin-1 beta; IL-6, interleukin-6; TNF, tumor necrosis factor.

BALF was collected, and the total cell numbers were counted to examine the infiltrated immune cell types in the lungs. OVA induced an increase in the total number of infiltrated cells compared to that in the control group, which was reversed by treatment with both 100 and 200 mg/kg of CKD-497 in BALF (Figure 4A). Differentiated inflammatory cells were also counted, and treatment with both 100 and 200 mg/kg CKD-497 inhibited OVA-induced increases in total cells and macrophages. However, the administration of 200 mg/kg of CKD-497, but not 100 mg/kg, inhibited the OVA-induced increase in neutrophils in the BALF (Figure 4A). The effect of CKD-497 on the production of inflammatory cytokines, such as IL-1β, IL-6, and TNF-α, was further confirmed. Serum samples were analyzed using ELISA, revealing significantly increased cytokine levels after the OVA challenge compared to those in the control group. However, mice treated with CKD-497 exhibited a significant reduction in serum IL-1β, IL-6, and TNF-α levels (Figure 4B).

### 3.5 CKD-497 alleviates histological changes and immune cell infiltration in PM-treated mouse lung tissues

**FIGURE 5:**
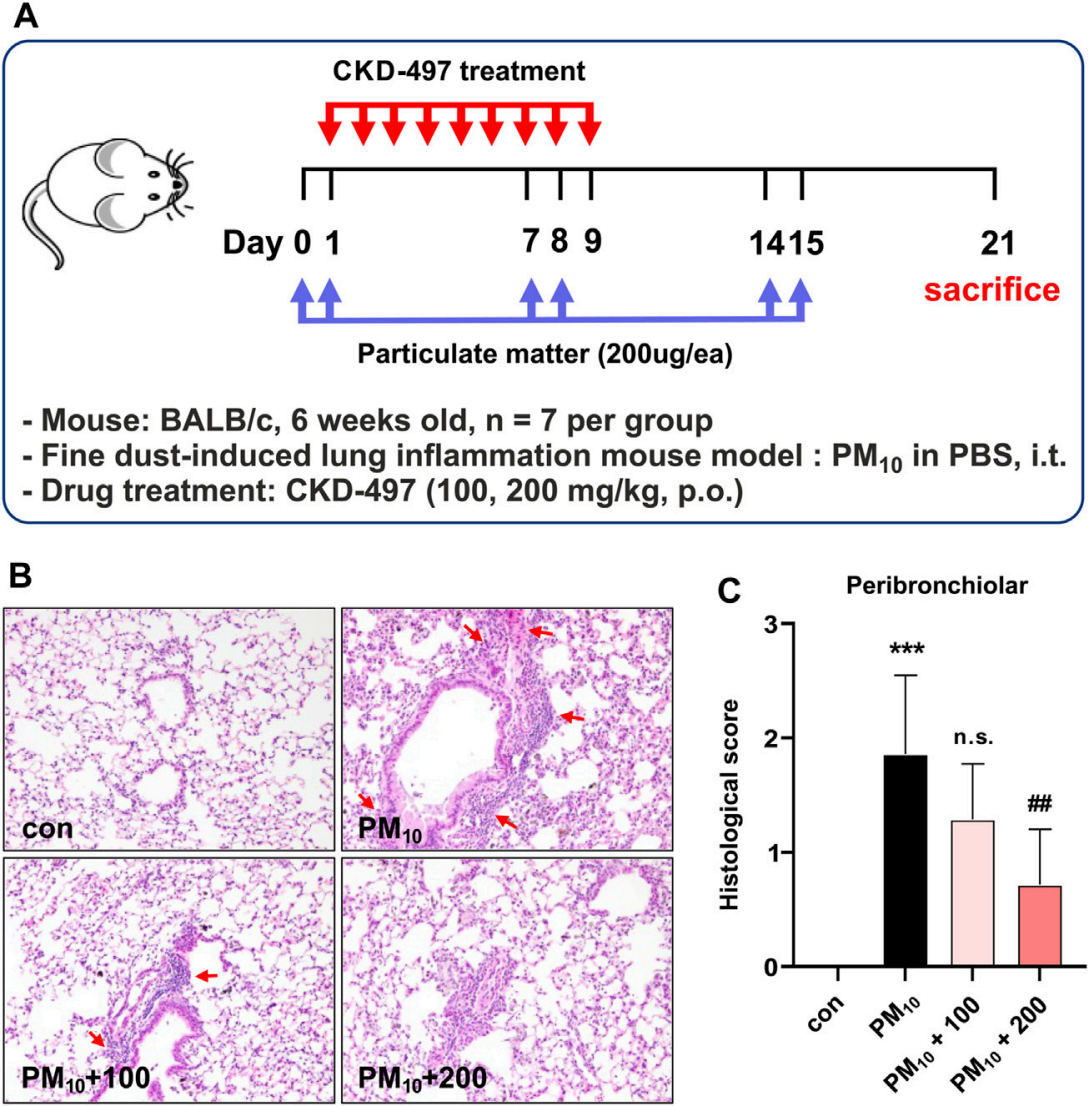
CKD-497 alleviates histological change and peribronchiolar inflammatory cell infiltration in PM_10_-treated mice lung tissue. **(A)** The experimental scheme of fine dust-induced mouse model of lung inflammation. BALB/c mice (n = 7) were treated intranasally with 200 μg PM_10_ on days 0, 1, 7, 8, 14, and 15. On day 21 post-treatment, mice were euthanized, and lung tissue samples were collected. **(B)** Representative hematoxylin-eosin-stained images of mouse lung sections. Lung injury was indicated by infiltration of immune cells. Interstitial hemorrhage is shown using red arrows. Magnification, ×200. **(C)** Histological scores are evaluated according to the grades as described in the Materials and methods. All data were analyzed using a one-way analysis of variance, followed by Dunnett’s multiple comparison test. ^***^ *p* < 0.001 vs. control. ^##^ *p* < 0.01 vs. PM_10_. Results are presented as mean ± SD values.

The effects of CKD-497 were then evaluated on a PM-induced airway inflammation mouse model. Mice were intratracheally exposed to PM_10,_ with or without oral CKD-497 treatment (Figure 5A). H&E-stained lung sections were microscopically observed to analyze the histopathological changes in each group. The control group exhibited normal alveolar morphology, whereas the PM_10_-treated group exhibited alveolar wall thickening accompanied by structural damage. However, CKD-497 treatment attenuated the PM_10_-induced immune cell infiltration and alveolar thickening (Figure 5B). Histological scores were also evaluated to quantify the damage (Figure 5C). The results indicated that 200 mg/kg of CKD-497 reversed the pathological changes that occurred in the lung tissue after exposure to PM_10_. These results suggest that PM_10_ can induce airway inflammation, and CKD-497 exhibits a significant protective effect in PM-associated lung tissue injury.

### 3.6 Effects of CKD-497 on immune cell infiltration and cytokine levels in the BALF and serum

**FIGURE 6:**
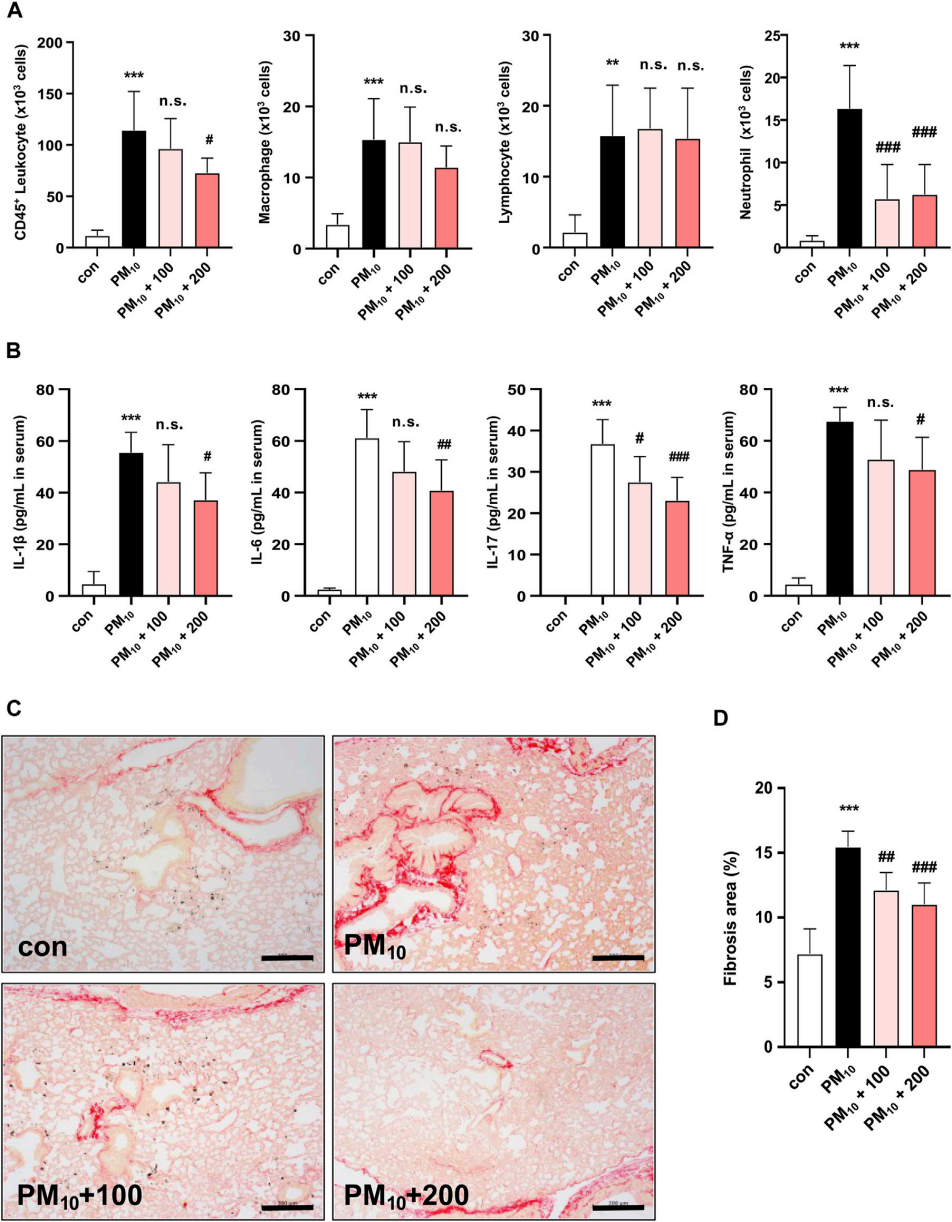
Histological analysis of the effects of CKD-497 on the severity of lung injury and fibrosis after PM_10_ induction. PM_10_ exposure triggered lung injury and enhanced the infiltration of inflammatory cells into the BALF. **(A)** Quantification of the number of CD45^+^ leukocytes, macrophages, lymphocytes, and neutrophils in the BALF. **(B)** ELISA was performed to detect the levels of IL-1β, IL-6, IL-17, and TNF-α in the serum of mice exposed to PM_10_. **(C)** Representative Sirius Red-stained images of mouse lung sections. Picrosirius Red staining was used to evaluate PM_10_-induced fibrosis in the lung tissues. Scale bar: 200 μm. **(D)** Percentage of the fibrotic area was calculated as the ratio of the area covered by the Picrosirius Red-positive lung area to the total lung area. All data were analyzed using a one-way analysis of variance, followed by Dunnett’s multiple comparison test. ^***^ *p* < 0.001 vs. control. ^***^ *p* < 0.001 vs. control. ^###^ *p* < 0.001, ^##^ *p* < 0.01 vs. PM_10_. The results are presented as mean ± SD values. BALF, bronchoalveolar lavage fluid; IL, interleukin; TNF, tumor necrosis factor.

The number of CD45^+^ leukocytes, macrophages, lymphocytes, and neutrophils was significantly increased in PM_10_-treated mice compared to those in control mice. Treatment with 200 mg/kg of CKD-497 significantly inhibited PM_10_-mediated increase in CD45^+^ leukocytes and neutrophils but did not affect the number of macrophages and lymphocytes. Notably, PM_10_-mediated increase in neutrophils was inhibited even with treatment with 100 mg/kg of CKD-497 (Figure 6A). Moreover, serum was collected to investigate the effect of CKD-497 on the production of inflammatory cytokines, such as IL-1β, IL-6, IL-17, and TNF-α, using ELISA. Serum cytokine levels were significantly higher in the PM_10_ group than in the control group. However, mice treated with CKD-497 exhibited significant reduction in serum IL-1β, IL-6, IL-17, and TNF-α levels (Figure 6B). Notably, the airways exhibited increased fibrillary collagen deposition after PM exposure, which was alleviated after treatment with CKD-497 (Figures 6C, D). These results suggest that CKD-497 has an anti-fibrotic efficacy by inhibiting PM-induced immune cell infiltration and secreted cytokines production.

## 4 Discussion

Allergic asthma is induced by intraperitoneal sensitization and intranasal challenge with OVA. The OVA-induced group exhibits infiltration of immune cells, goblet cell hyperplasia, and alveolar wall thickening (Ward et al., 2002). In the present study, we confirmed that CKD-497 can improve OVA-induced allergic asthma, PM-induced airway inflammation, and lung fibrosis, demonstrating the therapeutic potential of CKD-497 in the management of respiratory diseases. Airway mucus hypersecretion is a characteristic of many patients with asthma and clinically manifests as chronic cough and expectoration (Postma and Kerstjens, 1998; Subhashini et al., 2016). Our previous study demonstrated the beneficial effects of CKD-497 for cough inhibition using a guinea pig cough model (Chae et al., 2020). In an LPS-induced rat model, mucociliary clearance, a key factor in many chronic airway diseases, was increased by treatment with CKD-497. These results suggest that reduction in goblet cell hyperplasia by CKD-497 may also have a positive effect on chronic cough and phlegm caused by asthma. Chronic exposure to fine dust exacerbates asthma and other respiratory disorders by inducing airway inflammation. In the present study, we investigated the protective effects of CKD-497 using a PM-induced airway inflammation mouse model. Based on a previous study, we selected intratracheal instillation as the administration method to rapidly and accurately deliver PM to the trachea (Lee et al., 2023). As expected, PM_10_ aggravated airway inflammation and fibrosis, as demonstrated by the changes in cytokine levels, pathologic findings, and airway hyperresponsiveness. In the PM group, analysis of BALF revealed increases in the numbers of total leukocytes, including macrophages, lymphocytes, and neutrophils (Van Hoecke et al., 2017; Kalidhindi et al., 2021). Compared to the PM-only group, treatment with CKD-497 (100 and 200 mg/kg) in PM-exposed groups did not affect macrophages and lymphocytes, and only affected neutrophils. Neutrophilic inflammation is crucial in many pulmonary diseases, such as asthma, COPD, and cystic fibrosis (Jasper et al., 2019). In general, neutrophils constitute approximately 70% of leukocytes, represent a key component of the innate immune system, and are the first immune cells recruited to the lung upon pathogens infection (Rosales, 2018). Patients with airway diseases exhibit enhanced and destructive neutrophilic inflammation (Jasper et al., 2019). Similarly, in the present study, CKD-497 reduced the levels of PM-induced proinflammatory cytokines, including IL-1β, IL-6, IL-17, and TNF-α, whose overproduction leads to cell death and lung injury. IL-1β disrupts the barrier function between lung epithelial cells and pulmonary vascular endothelial cells. TNF-α and IL-6 trigger many innate immune cells, such as neutrophils, macrophages, and Th1 cells, thus amplifying inflammation and inducing a switch from an acute to a chronic inflammatory state (Hirano, 2021). Moreover, in our study, the PM_10_ group exhibited significant fibrotic changes in the airway lumen and perivascular area on PAS staining pathology, which were reversed by treatment with CKD-497. Collectively, these results indicate that CKD-497 improved PM-induced lung inflammation by inhibiting neutrophil-mediated inflammation and downregulating serum-derived inflammatory factors in lung bronchioles.

In order to elucidate the potential molecular mechanisms of CKD-497, an *in vitro* cell model of LPS-induced inflammation was used. This entailed stimulation of NF-κB signaling upon LPS exposure, leading to its translocation into the nucleus and subsequent induction of the expression of inflammatory genes. In our previous study, CKD-497 inhibited inflammatory mediators, such as IL-8 and TNF-α, in LPS-treated mouse macrophages and TRPV-1-overexpressed human bronchial epithelial cells stimulated by capsaicin. Here, we confirmed that CKD-497 significantly reduces LPS-induced inflammation in macrophage cells by suppressing NF-κB signaling.

In conclusion, these data suggest that CKD-497 may be effective for preventing and treating allergic asthma and PM-induced respiratory inflammation.

## Data Availability

The original contributions presented in the study are included in the article/supplementary material, further inquiries can be directed to the corresponding author.

## Abbreviations

BALF, bronchoalveolar lavage fluid; CKD, Chong Kun Dang; OVA, ovalbumin, LPS, lipopolysaccharide, PM, particulate matter; COVID-19, coronavirus disease 2019, NF-κB, Nuclear factor-kappa B; IL-1β, interleukin-1 beta; IL-6, interleukin-6; TNF, tumor necrosis factor.
